# Idazoxan and Efaroxan Potentiate the Endurance Performances and the Antioxidant Activity of Ephedrine in Rats

**DOI:** 10.3390/medicina57030194

**Published:** 2021-02-25

**Authors:** Gabriela Rusu-Zota, Alexandra Burlui, Elena Rezus, Luminita Paduraru, Victorita Sorodoc

**Affiliations:** 1 Department of Pharmacology-Algesiology, Faculty of Medicine, University of Medicine and Pharmacy ‘Grigore T. Popa’, Universitatii St. no. 16, 700115 Iasi, Romania; rusu.i.gabriela@umfiasi.ro; 2 Department of Rheumatology and Physiotherapy, Faculty of Medicine, University of Medicine and Pharmacy ‘Grigore T. Popa’, Universitatii St. no. 16, 700115 Iasi, Romania; elena.rezus@umfiasi.ro; 3 Department of Mother and Child Care (Neonatology), Faculty of Medicine, University of Medicine and Pharmacy ‘Grigore T. Popa’, Universitatii St. no. 16, 700115 Iasi, Romania; luminita.paduraru@umfiasi.ro; 4 Department of Internal Medicine (Toxicology), Faculty of Medicine, University of Medicine and Pharmacy ‘Grigore T. Popa’, Universitatii St. no. 16, 700115 Iasi, Romania; victorita.sorodoc@umfiasi.ro

**Keywords:** imidazoline, idazoxan, efaroxan, ephedrine, exercise, oxidative stress, endurance

## Abstract

*Background and objectives:* The connections between the imidazoline system and multiple other neurotransmitter systems in the brain (adrenergic, dopaminergic, serotoninergic, glutamatergic, opioid) indicate the complexity of the mechanisms underlying motor activity and behavior. The aim of the present research was to investigate the effects of the combination of ephedrine (EPD) and imidazoline antagonists idazoxan (IDZ) and efaroxan (EFR) on the endurance performance in the treadmill test in rats. *Materials and Methods:* We used Wistar rats distributed as follows: Group 1 (Control) receiving distilled water 0.3 mL/100 g body weight; Group 2 (EPD) receiving 20 mg/kg ephedrine; Group 3 (EPD + IDZ) receiving 20 mg/kg ephedrine + 3 mg/kg idazoxan; Group 4 (EPD + EFR) receiving 20 mg/kg ephedrine + 1 mg/kg efaroxan. An additional group (C) of animals receiving 0.3 mL/100 g body weight distilled water (but not subjected to effort) was used. Endurance capacity was evaluated using a treadmill running PanLAB assay. The evaluation of the substances’ influence on oxidative stress was performed by spectrophotometric determination of superoxide dismutase (SOD) and glutathione peroxidase (GPX) activity. *Results:* Treatment with EPD-IDZ and EPD-EFR were correlated with a longer distance traveled on the belt and with a decrease in the necessary electric shocks to motivate the animal to continue running in the forced locomotion test. Additionally, an increase in the activity of antioxidant enzymes was found. *Conclusions:* Idazoxan and efaroxan potentiated the physical effort-related effects of ephedrine with regard to endurance capacity and antioxidant activity in rats.

## 1. Introduction

First described in 1984, imidazoline receptors (I_1_, I_2_, I_3_) exhibit high affinity binding sites for various compounds with imidazole structure. I_1_ receptors mediate sympathetic inhibitory actions of imidazoline derivatives (decreasing blood pressure), I_2_ receptors modulate central monoamine levels and activate the hypothalamic-pituitary-adrenocortical axis, and I_3_ receptors regulate the insulin secretion from beta cells of the pancreatic islets of Langerhans [1].

Four endogenous ligands of imidazoline receptors have been characterized, agmatine (a decarboxylated arginine) being the most well-known and studied. Agmatine also acts as a ligand for alpha-2 adrenergic receptors as an antagonist of glutamate on NMDA (N-methyl-D-aspartate) receptors modulating the nitric oxide neuronal synthase activity [2]. Three other known endogenous ligands are harmane and harmalane (varieties of beta-carbolines) and robotide (an imidazole acetic acid derivative) [3].

The currently available literature indicates that imidazoline receptors are involved in various processes such as cell proliferation and adhesion, body fat regulation, inflammation, neuroprotection and neuropsychiatric diseases (such as depression and epilepsy) as well as cancer [3].

The cerebral imidazoline system is involved in multiple functions of the central nervous system, especially in behavioral modulation. Numerous experimental, clinical and pathological studies have highlighted the interrelationships between imidazoline, dopaminergic, adrenergic, glutamatergic, and opioid systems. This may explain the influence of various agents which use the imidazoline receptor pathway in the development and progression of cognitive disorders, behavioral disturbances and motor disorders [4].

Whereas different imidazoline agonists have been studied to date, few exhibit a selective action on the imidazoline receptors. Furthermore, certain potent imidazoline antagonists remain under investigation in clinical trials. The 2-(2,3-dihydro-1,4-benzodioxine-2-yl)-4,5-dihydro-1H-imidazole derivative idazoxan acts as an antagonist on both alpha-2 adrenergic and imidazoline receptors. It is now under investigation in different conditions such as depression, schizophrenia and certain types of psychosis especially as adjuvant therapy for antipsychotic medication. Nonetheless, significant results have not been reported to date [5,6]. Experimental studies have shown that systemic administration of 1 mg/kg body weight of idazoxan may lead to an intensification of the global motor behavior by increasing the horizontal activity, the number of rearing as well as the stereotype movements in the actimeter test in rats [7].

The 2-(2-ethyl-2,3-dihydro-1-benzofuran-2-yl)-4,5-dihydro-1H-imidazole derivative efaroxan is a highly selective alpha-2 adrenergic and I_1_ imidazoline receptor blocker [3]. Immunohistochemical studies and electrophysiological investigations have described the neuroprotective effects of both idazoxan and efaroxan in rats with quinolinic acid-induced brain lesions [8].

A new (+) 2-(ethyl-2,3-dihydrobenzofuranyl)-2-imidazoline-dexefaroxan derivative, the (+) efaroxan enantiomer with potent and selective antagonistic activity on the alpha-2 adrenergic receptors has demonstrated facilitatory influence on cognitive functions in different behavioral studies performed on lab animals [9,10,11]. Moreover, it exhibits neuroprotective effects on devascularization-induced neurodegeneration, ameliorates structural changes in the hippocampus and opposes the cognitive deficit induced by cerebral ischemia in rats [12,13].

The main objective of the present research was the experimental investigation of ephedrine combined with the imidazoline antagonists idazoxan and efaroxan on the endurance performance in the treadmill test in rats.

## 2. Materials and Methods

Substances. Idazoxan, efaroxan, ephedrine, and distilled water were purchased from Sigma-Aldrich Co, Germany. All solutions were prepared extemporaneously in distilled water.

Experimental animals. The laboratory animals were housed in plastic cages under standard laboratory conditions (constant temperature of 23.0 ± 1.0 °C, relative humidity 55–65%, and a 12-h artificial light/dark regimen), with standard granulated food and water ad libitum, except during the time of the experiments.

Protocol. A total of 4 groups of 6 male white Wistar rats (10–14 weeks old, 200–250 g each) were used in the experiment, treated intraperitoneally with the same volume, as follows:

Group 1 (coded Control): distilled water 0.3 mL/100 g body weight;

Group 2 (coded EPD): 20 mg/kg body weight ephedrine;

Group 3 (coded EPD + IDZ): 20 mg/kg ephedrine + 3 mg/kg body weight idazoxan;

Group 4 (coded EPD + EFR): 20 mg/kg ephedrine + 1 mg/kg body weight efaroxan.

The evaluation of effort capacity after administration of investigated substances was done using the treadmill test (PanLAB apparatus; Barcelona, Spain) in rats. The assay consisted of an automatic rolling belt with an adjustable speed and inclination angle allowing forced exercise running and truthful evaluation of physical endurance in laboratory animals [14,15]. The device was equipped with a stimulating grid able to generate electric shocks. Mild electric shocks were applied through the grill in order to force the animal to keep moving when fatigued.

This experimental model is a functional test that allows the exploration of spontaneous motor activity, motor coordination and of endurance performance of the lab animal under physical stress conditions [16]. The determinations were done over the same time interval (between 10.00 a.m. and 2.00 p.m.) in order to eliminate diurnal interferences. We set the following experimental parameters: intensity = 0.4 mA, speed = 40 cm/s and time = 15 min. We recorded the run distance, the number of stops near the grill area (which corresponds to the number of electric impulses the animal needed to receive in order to keep moving) and the time needed for applying mild electric shocks.

The sympathomimetic drug ephedrine has been demonstrated to improve physical performance and promote endurance in animals as well as humans in case of muscle fatigue [17]. In the present experimental model of forced locomotion, we tested the following premises:

- a decrease in the time interval for applying electric shocks or increasing the number of electric shocks corresponding to an increase of resistance to effort by the tested drug(s);
- on the contrary, prolonging the time duration of applying electric shocks or the number of electric shocks needed to be applied to the animal considered to be correlated to a decrease of resistance to effort induced by the investigated drug(s);
- the longer distance run by the animal on the belt during the recorded time indicating an increase of resistance to effort and a stimulation of motor activity by the tested drug(s);
- within the same experimental conditions, a shorter distance run by the animal on the belt during the recorded time corresponds to a diminution of resistance to effort, thus indicating a reduction of motor activity induced by the investigated drug.

The evaluation of the substances’ influence on oxidative stress was assessed by spectrophotometric determination of superoxide dismutase (SOD) and glutathione peroxidase (GPx) activity. The experiments were performed using the similar four groups of male white Wistar rats (200–250 g body weight) subjected to forced effort, and one additional group (coded C) with animals receiving 0.3 mL/100 g body weight distilled water but not subjected to effort.

Blood samples were collected after administration of the tested substances and after the animals were subjected to the stress test. Following collection in EDTA tubes (ethylenediaminetetraacetic acid), the samples were centrifuged at 4000 rpm for 10 min and the plasma was stored at −20 °C until analysis. The following oxidative stress parameters were determined: SOD (a natural antioxidant enzyme that detoxifies the superoxide radical, reducing its cellular disruptive effects) and GPx (an antioxidant enzyme that catalyzes the reduction of hydrogen peroxide by capturing and inactivating hydrogen and lipid peroxides). These enzymes are crucial antioxidant markers with important protective cellular action against oxidative stress [18,19].

Statistical analysis. We performed the statistical analysis of the data using IBM SPSS version 17 for Windows (New York, United States of America). The numerical results were expressed as mean +/− standard deviation (SD). We applied the ANOVA test (ANalysis Of VAriance) in order to examine the differences between groups, followed by the *post-hoc* Neumann-Keuls test. The statistical significance threshold was set at *p* ≤ 0.05.

Ethics approval. The study protocol was approved by the Ethics Committee on Research of the ‘Grigore T. Popa’ University of Medicine and Pharmacy for animal care and use (1/31.10.2013) and was in agreement with the EU directive 2010/63/EU pertaining to the handling of laboratory animals [20]. Each animal was used only once and the duration of the experiments was kept as short as possible in order to minimize their suffering. For ethical reasons, all rats included in the study were sacrificed at the end of the experiment.

## 3. Results

### 3.1. Evaluation of Endurance Capacity

**Figure 1:**
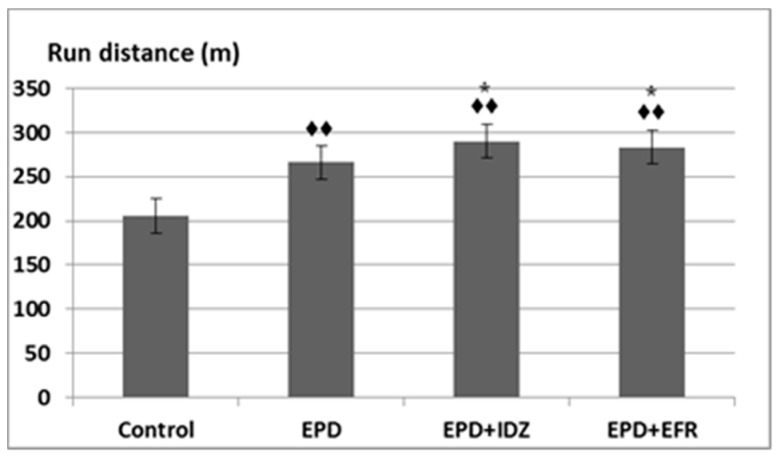
The effort test—the effects of ephedrine + idazoxan (EPD + IDZ) combination, respectively efaroxan (EFR), on the run distance in the treadmill test. Each value corresponds to the mean ± SD of run distance for 6 animals (♦♦ *p* < 0.01 vs. Control; * *p* < 0.05 vs. EPD group).

The parameters recorded during the sessions were the following: run distance, number and delivery time of electric shocks. During the time needed for the experiments, EPD determined the animals to run a longer distance on the belt (266.33 ± 21.07 m), significantly different compared to the Control group (205.67 ± 25.11 m) in the treadmill test (Figure 1).

Administration of the EPD + IDZ combination led to a longer distance run by the animals (290.17 ± 18.26 m), statistically significant compared to the Control group, but also compared to the EPD group in the effort test in rats (Figure 1). The treatment with the EPD + EFR combination resulted in an increase of the run distance (283.17 ± 16.59 m) which was statistically significant compared to the Control group as well as the EPD group in the locomotion test (Figure 1). These effects were less intense compared to those of EPD + IDZ on the distance run by the animal during the same time interval in this experiment.

**Figure 2:**
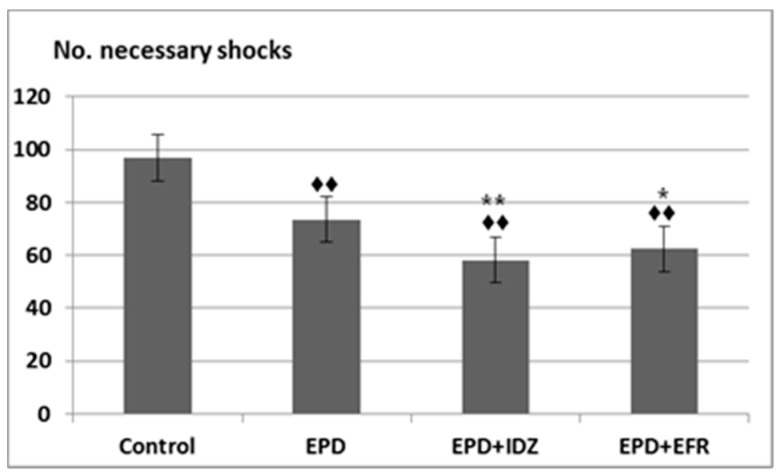
The effort test—the effects of EPD + IDZ combination, respectively EFR, on the number of necessary electric shocks applied to the animals to keep running on the belt in the treadmill test. Each value corresponds to the mean ± SD of applied shocks number for 6 animals (♦♦ *p* < 0.01 vs. Control; * *p* < 0.05, ** *p* < 0.01 vs. EPD group).

The administration of EPD led to a decrease in the number of necessary electric shocks (73.50 ± 10.93). This difference was statistically significant compared to the Control group (96.83 ± 10.15) in the effort test (Figure 2). Furthermore, the intraperitoneal injection of EPD + IDZ was linked to a notable reduction of the number of electric shocks (58.17 ± 8.18), statistically significant compared to Controls as well as the group that received EPD alone. The use of EPD + EFR was correlated with less electric stimulations needed (62.33 ± 11.54), statistically significant compared to the group that received distilled water, respectively to EPD alone in the forced locomotion test (Figure 2). Its effects on the number of electric shocks were less accentuated compared to those of EPD + IDZ in the same session of the experiment.

**Figure 3:**
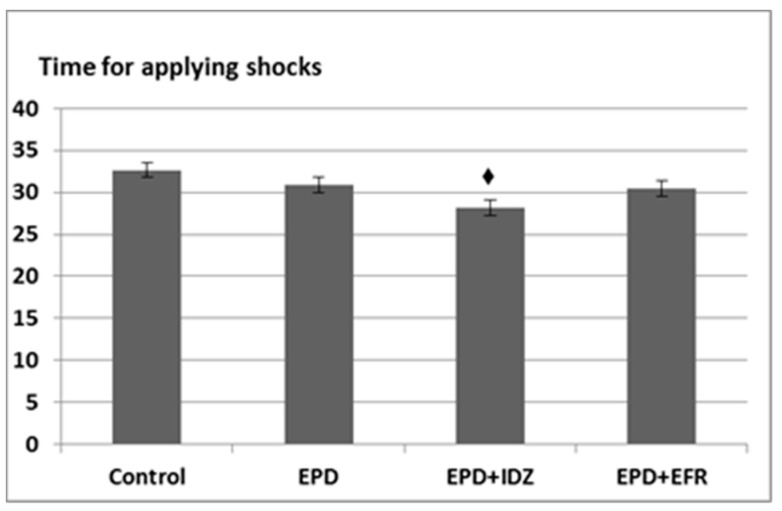
The effort test—the effects of the EPD + IDZ combination, respectively EFR on the period (s) for applying electric shocks in the treadmill test. Each value corresponds to the mean ± SD of period for applying the electric shocks for 6 animals (♦ *p* < 0.05 vs. Control).

Intraperitoneal administration of EPD lead to a decrease in the period of time for applying shocks (30.83 ± 3.06 s), which was, however, not statistically significant compared to the Control group (32.67 ± 6.83 s) in the effort test (Figure 3).

The treatment with EPD + IDZ induced a decrease in the interval of time for applying electric stimulation (28.17 ± 2.32 s), statistically significant compared to the Control group. The administration of EPD + EFR resulted in a slight diminution (30.50 ± 2.74 s) of the time period for applying the electric shocks, but non-significant compared to the Control group (Figure 3).

### 3.2. Evaluation of Oxidative Stress

The results of oxidative stress parameters were recorded for the rats treated with EPD, EPD + IDZ, EPD + EFR and forced to run in the treadmill test. In response to endurance training in rats, free radicals are produced due to oxidative stress (22). The investigation of SOD and GPx activity changes provides the evaluation of the influence of administered substances on oxidative stress.

A significant decrease of SOD values (76.45 ± 6.31 U/mL) could be observed in rats from the Control group subjected to effort, statistically significant compared to C group without effort (102.42 ± 5.80 U/mL).

**Figure 4:**
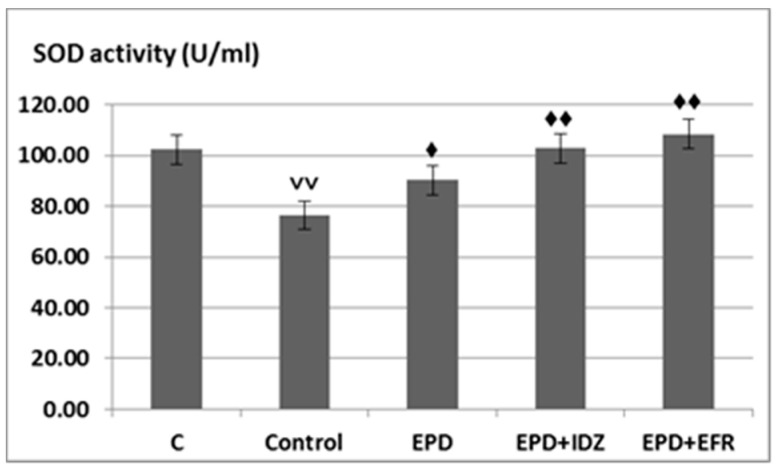
The effects of EPD + IDZ combination, respectively EFR on the SOD activity. Each value corresponds to the mean ± SD of SOD levels for 6 animals (♦ *p* < 0.05, ♦♦ *p* < 0.01 vs. Control group; ˅˅ *p* < 0.01 vs. Control group).

The use of EPD resulted in an accentuation of SOD activity (90.22 ± 7.32 U/mL), statistically significant compared to the Control group in the treadmill test (Figure 4).

The administration of EPD + IDZ was correlated with an increase in SOD activity (102.90 ± 6.08 U/mL), statistically significant compared to the Control group subjected to forced locomotion. The treatment with EPD + EFR led to an intense increase of SOD values (108.40 ± 4.92 U/mL), statistically significant compared with the Control group forced to effort, the effects being more intense than those of EPD + IDZ in the treadmill test (Figure 4).

**Figure 5:**
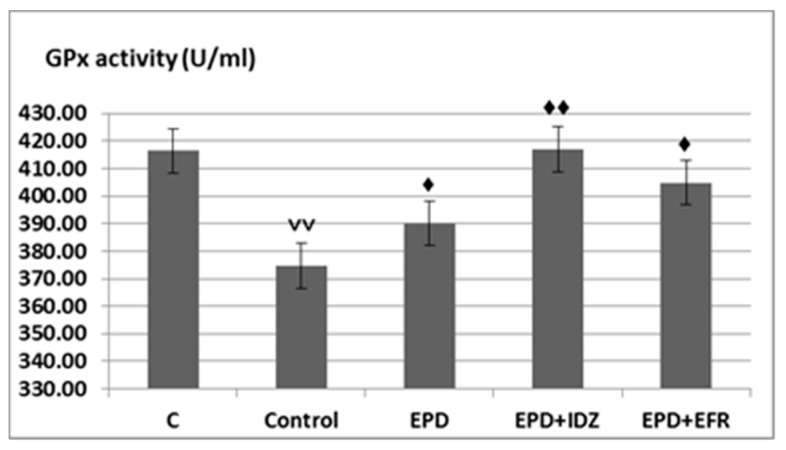
The effects of EPD + IDZ combination, respectively EFR on the GPx activity. Each value corresponds to the mean ± SD of GPx levels for 6 animals (♦ *p* < 0.05, ♦♦ *p* < 0.01 vs. Control; ˅˅ *p* < 0.01 vs. C group).

An important decrease in GPx levels was observed (347.72 ± 26.04 U/mL) in the Control group, the rats which were subjected to running effort in the treadmill test, statistically significant compared to their counterparts (416.44 ± 18.28 U/mL) (Figure 5).

Treatment with EPD led to a more intense activity of GPx (390.03 ± 35.60 U/mL), statistically significant compared to the Control group in the forced locomotion test in rats (Figure 5).

The combined administration of EPD and IDZ was associated with a statistically significant accentuation of the GPx activity (416.96 ± 36.25 U/mL), compared to the group treated with distilled water in the treadmill test (Figure 5).

The use of EPD + EFR resulted in an increase of GPx levels (404.90 ± 45.35 U/mL), statistically significant compared to the Control group in this experimental behavioral model in rats. The effects of EPD + EFR on GPx values reduction were more pronounced than those returned by EPD + IDZ in the treadmill test in rats (Figure 5).

## 4. Discussion

The currently available literature indicates that the imidazoline receptors expressed centrally as well as peripherally mediate a variety of physiological and pathological processes. However, the mechanisms underlying the imidazoline receptor pathway are still to be clarified. The existence of a link between imidazoline signaling and multiple other neurotransmitter systems in the brain (the adrenal, dopaminergic, serotoninergic, glutamatergic, opioid systems, and others) indicates that such nervous system disturbances as memory loss, learning impairment, behavioral or motor issues may develop through highly complex mechanisms [4,21,22,23,24,25].

The results reported in literature concerning the pharmacodynamic effects of substances acting on the imidazoline receptors are few and frequently discrepant [26]. Further investigations on the involvement of these substances in processes such as behavior, memory, or motor activity may be the starting point for acquiring new information about the physio-pathological mechanisms that define spontaneous behavior and cognitive impairment [27].

Various neuro-pharmacology studies have been focused on two ligands of the I_2_ receptors efaroxan and idazoxan, involved in modulation of behavior, cognitive functions and motor activity. Both substances are I_1_ and I_2_ antagonists but also act as alpha-2 adrenergic receptor antagonists [8,28,29]. Previous experimental research on lab animals have shown that efaroxan and idazoxan completely block the anti-compulsive effects of agmatine suggesting the involvement of imidazoline receptors in anxiety and obsessive-compulsive spectrum disorders [30].

The results reported by experimental research showed that imidazoline receptors could be therapeutically targeted for the treatment of depressive disorders, due to the fact that both efaroxan and idazoxan may block the anti-depressant-like effects of bupropion and also antagonize the synergic bupropion-agmatine combination [31].

Other experimental studies have demonstrated the involvement of imidazoline receptors in the inhibition of learning activities in rats [30]. The retrodialysis of idazoxan resulted in the enhancement of norepinephrine release induced by shocks applied on experimental animals’ basolateral amygdala [29].

In the present study we used the forced locomotion test to evaluate rats’ endurance capacity after administration of ephedrine and idazoxan, respectively of ephedrine and efaroxan combinations. The physical effort assessment used in the experiment is one of the most appreciated tests evaluating the influence of the investigated substances’ effects on behavior and endurance capacity of rats subjected to forced locomotion. This experimental model is also a valuable method to elucidate the movement motivation and physical abilities of lab animals [16]. The treatment with ephedrine + idazoxan and ephedrine + efaroxan was correlated with a longer distance traveled on the belt and with a decrease in the necessary electric shocks applied for stimulating the animals to keep running. Our findings indicate that the association of imidazoline receptor antagonists may potentiate ephedrine-related endurance capacity, the influence of idazoxan being more intense than that of efaroxan over the same time interval in the experiment.

It is known that the stress induced by skeletal muscle contraction increases the generation of reactive oxygen species the products of which prompt the oxidation of nucleic acids, proteins and lipids, significantly reducing the antioxidant capacity and thus resulting in fatigue [17,32,33]. Subjecting the lab animals to forced physical effort leads to a production of free radicals evidenced by a marked decrease in SOD and GPx values [34,35,36,37]. In the Control group, we found lower SOD and GPx levels in the rats which were subjected to physical effort compared to those that did not participate in the treadmill test. These findings are consistent with literature data regarding the changes in GPx activity during physical effort [17,21,23]. The investigation of SOD and GPx activity allowed us to deduct the influence of the association between ephedrine and the imidazoline receptor antagonists on oxidative stress in lab animals.

Further experiments such as a swimming test could provide additional insight regarding the effects of efaroxan and idazoxan (alone or in combination with ephedrine) on laboratory animals’ behavior and physical performance, as well as oxidative stress. The doses of ephedrine, idazoxan and efaroxan used in our experiment are concordant with those applied in previously published research in the field. In this regard, the administration of ephedrine 20 mg/kg has been used in studies pertaining to exercise fatigue in rats, whereas 3 mg/kg idazoxan and 1 mg/kg efaroxan were administered in combination with agmatine to test their influence on locomotor activity following experimentally-induced spinal cord injury [38,39]. Moreover, similar doses of idazoxan and efaroxan were used in studies pertaining to cognitive functions and animal models of depression [40,41]. Nevertheless, the potentially dose-dependent relationships between ephedrine + idazoxan and ephedrine + efaroxan combinations and their impact on rats’ physical performance, cognitive issues and behavior remain to be fully described. 

Our study demonstrated that treatment with ephedrine + idazoxan and ephedrine + efaroxan was correlated with an increase in SOD and GPx activity, suggesting the protective effect of these combinations against oxidative stress [34,35,36,37]. Moreover, we found that idazoxan boosted the ephedrine-induced increase of SOD values, while efaroxan promoted those of GPx.

## 5. Conclusions

Our experimental study showed that the addition of imidazoline receptor antagonists idazoxan and efaroxan boosted the effects of ephedrine in terms of increasing locomotor activity and endurance capacity in the treadmill test in rats. Nevertheless, the effects of the ephedrine and idazoxan combination were more intense than those found in the ephedrine and efaroxan group with respect to the lab animals’ performance in the forced locomotion test. Furthermore, idazoxan and efaroxan enhanced the antioxidant properties of ephedrine in the experimentally-induced stress in rats subjected to forced physical effort in the treadmill running test.

## Data Availability

Not applicable.
